# The Effect of Recruitment Maneuvers on Cerebrovascular Dynamics and Right Ventricular Function in Patients with Acute Brain Injury: A Single-Center Prospective Study

**DOI:** 10.1007/s12028-024-01939-x

**Published:** 2024-02-13

**Authors:** Filippo Sanfilippo, Agnieszka Uryga, Lorenzo Ball, Denise Battaglini, Ida Giorgia Iavarone, Peter Smielewski, Erta Beqiri, Marek Czosnyka, Nicolò Patroniti, Chiara Robba

**Affiliations:** 1 Department of General Surgery and Medico-Surgical Specialties, School of Anaesthesia and Intensive Care, University of Catania, Catania, Italy; 2 Department of Biomedical Engineering, Wroclaw University of Science and Technology, Wrocław, Poland; 3 Department of Surgical Sciences and Integrated Diagnostics (DISC), University of Genoa, Genoa, Italy; 4 Anesthesia and Intensive Care, IRCCS Policlinico San Martino, Largo Rosanna Benzi, 16100 Genoa, Italy; 5 Brain Physics Laboratory, Division of Neurosurgery, Department of Clinical Neurosciences, University of Cambridge, Cambridge, UK

**Keywords:** Cerebral perfusion, Intracranial pressure, Mean arterial pressure, Intrathoracic pressure, Cerebral autoregulation, Echocardiography

## Abstract

**Background:**

Optimization of ventilatory settings is challenging for patients in the neurointensive care unit, requiring a balance between precise gas exchange control, lung protection, and managing hemodynamic effects of positive pressure ventilation. Although recruitment maneuvers (RMs) may enhance oxygenation, they could also exert profound undesirable systemic impacts.

**Methods:**

The single-center, prospective study investigated the effects of RMs (up-titration of positive end-expiratory pressure) on multimodal neuromonitoring in patients with acute brain injury. Our primary focus was on intracranial pressure and secondarily on cerebral perfusion pressure (CPP) and other neurological parameters: cerebral autoregulation [pressure reactivity index (PRx)] and regional cerebral oxygenation (rSO_2_). We also assessed blood pressure and right ventricular (RV) function evaluated using tricuspid annular plane systolic excursion. Results are expressed as the difference (Δ) from baseline values obtained after completing the RMs.

**Results:**

Thirty-two patients were enrolled in the study. RMs resulted in increased intracranial pressure (Δ = 4.8 mm Hg) and reduced CPP (ΔCPP = −12.8 mm Hg) and mean arterial pressure (difference in mean arterial pressure = −5.2 mm Hg) (all *p* < 0.001). Cerebral autoregulation worsened (ΔPRx = 0.31 a.u.; *p* < 0.001). Despite higher systemic oxygenation (difference in partial pressure of O_2_ = 4 mm Hg; *p* = 0.001) and unchanged carbon dioxide levels, rSO_2_ marginally decreased (ΔrSO_2_ = −0.5%; *p* = 0.031), with a significant drop in arterial content and increase in the venous content. RV systolic function decreased (difference in tricuspid annular plane systolic excursion = −0.1 cm; *p* < 0.001) with a tendency toward increased RV basal diameter (*p* = 0.06). Grouping patients according to ΔCPP or ΔPRx revealed that those with poorer tolerance to RMs had higher CPP (*p* = 0.040) and a larger RV basal diameter (*p* = 0.034) at baseline.

**Conclusions:**

In patients with acute brain injury, RMs appear to have adverse effects on cerebral hemodynamics. These findings might be partially explained by RM’s impact on RV function. Further advanced echocardiography monitoring is required to prove this hypothesis.

**Supplementary Information:**

The online version contains supplementary material available at 10.1007/s12028-024-01939-x.

## Introduction

Patients with acute brain injury (ABI) often require management in the intensive care unit (ICU) with invasive mechanical ventilation (MV), which presents unique challenges in this population [1]. Attention should be paid to ensuring that the ventilatory strategies, namely, the choice of tidal volume (TV), plateau pressure, and positive end-expiratory pressure (PEEP), do not compromise brain function while protecting the lungs [2–4]. Achieving this balance may be complex and, in some cases, a protective MV regime is not feasible because the priority is to maintain adequate carbon dioxide (CO_2_) values to prevent secondary brain injury [4].

Moreover, MV settings may profoundly impact intracranial pressure (ICP), not only as a result of CO_2_ change. MV may exert substantial hemodynamic effects, particularly by impeding venous return and/or excessively straining the right ventricle (RV) [5, 6]. Traditionally, high PEEP levels have been discouraged in patients with ABI, as PEEP itself may reduce the venous return to the RV and elevate pulmonary vascular resistances (PVRs) [7]. This can result in jugular congestion, potentially elevating ICP [8]. However, in some patients, higher PEEP levels may improve clinical conditions if it achieves recruitment of atelectatic lung parenchyma and if it reduces ventilatory-perfusion mismatch in areas suffering from pulmonary contusions (associated with concomitant traumatic injuries) [5].

Recruitment maneuvers (RMs) refer to the application of higher levels of PEEP and/or TV using various protocols to improve lung recruitment, thereby expanding the parenchyma viable for gas exchanges [9]. The use of RMs has been deeply investigated in patients with acute respiratory distress syndrome, but the effects on patient outcome have not been encouraging [9]. Multiple reasons have been proposed to explain the lack of success, suggesting that only a subset of patients with acute respiratory distress syndrome may derive benefit from RMs.

The most recent guidelines on MV for patients with ABI did not provide any recommendations on the use of RMs because of both unclear evidence on their efficacy and safety and lack of consensus among experts [10]. From a physiological perspective, it is plausible that a successful RM with longstanding lung recruitment would positively influence the RV for the reduction of PVRs together with an improvement in gas exchanges. Such effects may be desirable in patients with ABI. However, during RM application, patients may experience venous congestion and considerable strain may be imposed on the RV with consequent hemodynamic repercussions. Consequently, in patients with ABI, RMs could transiently induce adverse effects on ICP because of the cardiovascular impact of these maneuvers themselves.

We performed a single-center, prospective study to investigate the impact of RMs on multimodal monitoring parameters, primarily focusing on ICP but also on cerebral autoregulation and cerebral oxygenation in patients with ABI. Secondly, we aimed to evaluate the effects of RMs on the RV function, assessing the interplay between changes in RV function, hemodynamics, and cerebral variables during RMs.

## Methods

A single-center, prospective, observational study was conducted at the neurointensive care unit at San Martino Policlinic Hospital, Istituto di Ricovero e Cura a Carattere Scientifico for Oncology and Neurosciences, Genoa, Italy. The study was approved by the local ethics review board (Comitato Etico Regione Liguria, protocol number Comitato Etico Regione Liguria: 23/2020) and conducted according to the Strengthening the Reporting of Observational Studies in Epidemiology statement for observational cohort studies (Additional file 1: Supplementary Table S1). Patients were enrolled from February 1, 2021, to May 1, 2023.

### Inclusion criteria

We included adult patients (> 18 years old) admitted to the neurointensive care unit following ABI (defined as traumatic brain injury [TBI], intracranial hemorrhage [ICH], or subarachnoid hemorrhage [SAH]) and monitored with invasive ICP and multimodal neuromonitoring tools, including cerebral autoregulation (through the pressure reactivity index [PRx]) and near infrared spectroscopy (NIRS) for the noninvasive assessment of regional cerebral oxygenation (rSO_2_). We included patients sedated and ventilated on controlled MV, for which the treating physician gave clinical indications for a RMs within the first week of ICU stay. We excluded patients with the following: (1) high ICP values (> 22 mm Hg for at least 5 min) at the moment of the RMs or in the previous 48 h; (2) requirements for higher than tier 0 therapies for ICP management according to current guidelines [11] (tier 0 treatments include basic measures for controlling ICP as intubation and MV, serial evaluations of neurological status, head elevation, analgesia, and sedation just to achieve comfort, avoidance of fever); (3) left ventricular systolic dysfunction, defined as an ejection fraction below 45%; (4) presence of at least moderate heart valve disease; (5) hemodynamic instability, requiring high doses of vasoactive drugs defined as norepinephrine dosage above 0.30 µg/kg/min (or equivalents [12]) to obtain a cerebral perfusion pressure (CPP) of at least 60 mm Hg.

### Population and management

Patients were managed according to local protocols and current guidelines for the management of ICP in ABI [13, 14], using continuous infusion of propofol and MV was set using TV of 6–8 mL/kg of predicted body weight, aiming to maintain a plateau pressure < 27 cmH_2_O, as long as this setting allowed control of partial pressure of O_2_ (PaCO_2_) values (35–40 mm Hg). Midazolam was used only in the initial phase of ABI when ICP has usually more variability; however, no patients were on midazolam during RMs. In this population of patients with ABI, arterial blood pressure was continuously monitored in the radial or femoral artery (Baxter Healthcare CA; Sidcup, UK). The ICP was continuously monitored using an intraparenchymal probe or an external ventricular drain according to clinical indications. Cerebral autoregulation was estimated using PRx [15] in Intensive Care Monitor software (ICM+; Cambridge Enterprise, Cambridge, UK, https://icmplus.neurosurg.cam.ac.uk/). Briefly, PRx represents the moving Pearson correlation between the slow fluctuations in mean arterial pressure (MAP) and ICP; if the two parameters are positively correlated, this indicates altered autoregulation, whereas negative or low values of PRx (and in particular below 0.3 a.u.) indicate preserved autoregulation [16]. Cerebral oxygenation was measured with NIRS technology (Masimo O3 regional Oximetry monitor, Irvine, CA), using bilateral sensors placed in the forehead region, obtaining the total value of rSO_2_ as well as the variation in its components, arterial-oxyhemoglobin (ΔO_2_Hbi) and venous-deoxyhemoglobin (ΔHHbi), and the sum of the latter components (total hemoglobin).

### RMs

The decision to start RM was related to the clinician’s assessment and judgment. In our institution, the use of RM in patients with ABI is considered only as rescue therapy when patients present PaO_2_/FiO_2_ values < 150 mm Hg for more than 6 h with worsening clinical trajectory [17] and when the patients are considered potentially PEEP responders according to respiratory system mechanics and computed tomography or lung ultrasound findings. As for our local protocol, after optimizing lung protective strategies, we progressively increased the FiO_2_ to 100% before starting the RM, and this FiO_2_ was maintained until the end of the maneuver. The RM was applied through an escalating PEEP strategy; in particular, the TV was kept constant, and the PEEP was progressively increased up to maximal inspiratory pressure 35–40 cmH_2_O for 30 s (five breaths at each PEEP level), followed by decremental PEEP titration according to oxygen saturation of arterial blood, respiratory system mechanical properties, and hemodynamic parameters. The measurements to evaluate the neurological, respiratory, and hemodynamic impact of the RMs were taken at the achievement of the highest level of PEEP. As a safety measure, in case of increased ICP > 25 mm Hg, a bolus of mannitol or 5% hypertonic saline (100 ml) was administered as per our protocol. If the increase in ICP was recorded during the RM and was not promptly responding to the treatment (within 2 min), the maneuver itself was stopped and the patient was excluded. If a refractory increase in ICP happened after the full RM was completed, the patient was not excluded.

### Data collection and end points

Demographics and clinical data, information on neurological status at admission (as for Glasgow Coma Scale), ICU complications and ICU mortality were extracted by two independent operators through the electronic clinical notes and securely stored in a medical computer in a password protected Excel file. Neuromonitoring, clinical, and cardiovascular parameters were compared between two time points: at baseline before the RMs (T0), and at the end of RMs with the highest level of PEEP achieved (T1). All neuromonitoring indices were averaged in a moving time window of 10 s.

### Assessment of the RV function

We evaluated the repercussions of RMs on the RV to understand if changes in neuromonitoring parameters (if there were any) could be associated with the impact of RMs on the heart. A trained investigator (with more than 100 examinations performed in the last 2 years) performed basic monitoring of RV function with transthoracic echocardiography using the apical four-chamber view. In particular, we evaluated three parameters: (1) the RV systolic function, by means of tricuspid annular plane excursion (TAPSE) (in centimeters); (2) the RV dilatation, looking at its basal diameter (in centimeters); and (3) the presence of tricuspid regurgitation jet velocity (TRvel) (in centimeters per second) to describe the impact on PVRs. The reporting of echocardiographic variables in this study attempts to align with current recommendations for critical care echocardiography studies [18, 19]. However, because of the observational nature of the study, not all variables could be collected. Assessment of RV function was performed at T0 and T1.

### Statistical analysis

The assessment of data normality was conducted through the Shapiro–Wilk test. Given the absence of a normal distribution in the majority of the analyzed parameters and the presence of limited number of observations, nonparametric tests were employed. A *χ*^2^ test was used to compare the observed frequencies of categorical data in a contingency table. Wilcoxon signed-rank tests were used to compare values of different variables between T0 and T1 for our primary aim.

A first subgroup analysis was conducted separating population in two groups according to the median value of ΔCPP (in which ΔCPP was defined as CPP at T1 – CPP at T0) to identify the clinical characteristics of patients with a good or a poor CPP tolerance to the RMs. A poor tolerance to RM was defined as values of ΔCPP below the median. Similarly, a second subgroup analysis was performed focusing on cerebral autoregulation, splitting the population in two according to the median value of ΔPRx (in which ΔPRx was defined as PRx at T1 – PRx at T0) to identify the clinical characteristics of patients with a good or a poor PRx tolerance to the RMs. Considering the established cutoffs for definition of high ICP (> 22 mm Hg) and poor autoregulation (PRx > 0.3 a.u.), we conducted other two subgroup analyses. The Mann–Whitney *U*-test was applied to compare two independent subgroups and a rank-biserial correlation coefficient served as the measure of effect size. Correlation coefficients, along with their 95% confidence intervals, between variables were assessed using Spearman’s rank test. The matrix of Spearman was generated to explore the correlations between the cardiopulmonary variables and neurological parameters. Correlation coefficients less than 0.3 was interpreted as weak, more than 0.3 but less than 0.6 as moderate, more than 0.6 but less than 0.9 as strong, and more than 0.9 as excellent [20]. The rectangular outlines within the correlation matrix plot were determined by the results of hierarchical clustering. The significance level for all analyses was set at 0.05. Statistical analyses were conducted using STATISTICA 13 (Tibco, Palo Alto, CA) and R Statistical Software (v.4.0.2; R Foundation for Statistical Computing, Vienna, Austria) with the “ggstatsplot” package [21]. Data are presented in the format of median (first–third quartile), unless otherwise specified, and visualized with violin and boxplots.

## Results

### General characteristics

Over a period of 27 months, 32 patients (age 57 ± 31; women 38%) fulfilled the inclusion criteria. No patients experienced refractory increase in ICP during the RM requiring exclusion from the study. There were 54% of patients diagnosed with TBI, 28% with SAH, and 18% with ICH. Median Glasgow Coma Scale (GCS) at admission was 6 (3–9), eight patients (25%) died in the ICU, and 16 (50%) had a poor neurological outcome as evaluated by the Glasgow Outcome Scale Extended (GOSE 1–4). Half of nonsurvivors (*n* = 4) and half of poor neurological outcome patients (*n* = 8) had poor CPP tolerance to RMs. The majority of patients were supported by noradrenaline (*n* = 28, 88%) for optimization of CPP, with a mean dosage of 0.15 (0.05–0.20) µg/kg/min. Baseline characteristics of the population are shown in Table 1.

**Table 1:** Clinical patient characteristics

Parameter	Total group (*n* = 32)
Age (y)	57 (39–70)
BMI (a.u.)	25.3 (23.4–26.9)
Female sex, *n* (%)	12 (38)
Noradrenaline, *n* (%)	28 (87.5)
Noradrenaline, dosage (µg/kg/min)	0.15 (0.05–0.20)
Comorbidities^a^, *n* (%)	
Smoking	6 (19)
Hypercholesterolemia	1 (3)
Hypertension	7 (22)
Depression/anxiety and depression disorders	5 (16)
Kidney disease and hyperuricemia	2 (6)
Thyroid diseases	3 (9)
Cluster headache	1 (3)
Others	4 (13)
None	10 (32)
Type of acute brain injury, *n* (%)	
TBI	17 (54)
ICH	6 (18)
SAH	9 (28)
GCS	6 (3–9)
Abnormal pupillary reflex, *n* (%)	9 (28)
Marshall classification^b^	4 (3–5)
mFisher scale^c^	4
Days on MV	14 (10–19)
ICU length of stay	29 (21–39)
ICU mortality, *n* (%)	8 (25)
Noradrenaline support, *n* (%)	28 (88)
Noradrenaline dosage (µg/kg/min)	0.15 (0.05–0.20)
ICU GOSE	3 (2–4)
ICU complications^a^, *n* (%)	
Meningitis	1 (3)
Hydrocephalus	1 (3)
VAP	11 (35)
Infection	2 (6)
Candidemia	1 (3)
Sepsis	13 (41)
Brain death	1 (3)
Cerebral vasospasm	2 (6)
Epilepsies	2 (6)
Acute renal failure	1 (3)
None	9 (22)

Data are presented as either the median (with lower and upper quartile) or as the number of study participants (with the percentage of the group)

*BMI* body mass index, *GCS* Glasgow Coma Scale, *GOSE* Glasgow Outcome Scale Extended, *ICH* intracranial hemorrhage, *ICU* intensive care unit, *mFisher* modified Fisher scale, *MV* mechanical ventilation, *SAH* subarachnoid hemorrhage, *TBI* traumatic brain injury, *VAP* ventilator-associated pneumonia

^a^More than one is possible

^b^Only for patients with TBI

^c^Only for patients with SAH

### Effect of RMs on multimodal monitoring parameters

**Fig. 1:**
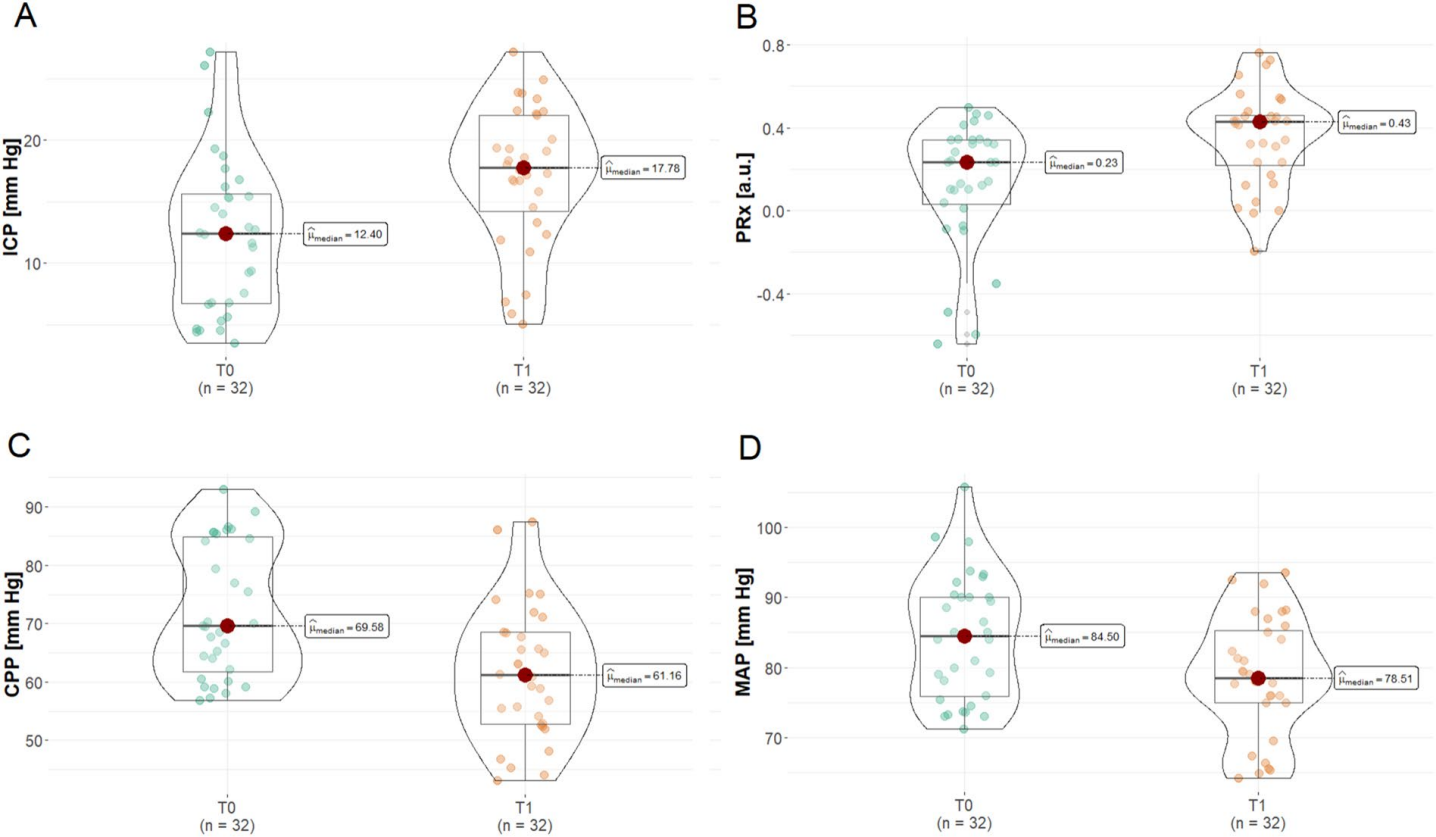
Violin and boxplots representing baseline (T0) and the effect of recruitment maneuver (RM) therapy (T1) on **A** intracranial pressure (ICP), **B** cerebral autoregulation measured with pressure reactivity index (PRx), **C** cerebral perfusion pressure (CPP), and **D** mean arterial blood pressure (MAP). Values are presented as median and interquartile range

Table 2 presents neurological parameters estimated at T0 and T1. In comparison with T0, ICP and PRx significantly increased at T1 (see also Fig. 1a, b). We also observed a significant reduction in both CPP and MAP at T1 (Fig. 1c, d). Furthermore, both rSO_2_ and ΔO_2_Hbi significantly decreased, whereas HHbi significantly increased at T1 in comparison with T0.

**Table 2:** Systemic and neuromonitoring data before (T0) and after (T1) recruitment maneuvers (RMs)

Parameter	T0	T1	*p* value	*r*_rb_	Δ change
ICP (mm Hg)	12.4 (6.7–15.8)	17.8 (13.9 to 22.1)	** < 0.001**	0.70	4.8 (2.5 to 8.2)
CPP (mm Hg)	69.6 (61.3–85.0)	61.2 (52.7 to 68.5)	** < 0.001**	0.73	− 12.8 (− 18.0 to − 3.7)
MAP (mm Hg)	84.5 (75.7–90.2)	78.5 (75.0 to 85.5)	** < 0.001**	0.60	− 5.2 (− 9.9 to 2.0)
PRx (a.u.)	0.23 (0.02–0.34)	0.43 (0.24 to 0.51)	** < 0.001**	0.61	0.31 (0.1 to 0.40)
SpO_2_ (%)	97.5 (96.0–98.0)	98.0 (96.7 to 100.0)	0.075	0.31	1.0 (− 1.0 to 2.0)
rSO_2_ (%)	60.6 (55.3–63.6)	59.0 (54.8 to 62.8)	**0.031**	0.38	− 0.5 (− 1.0 to 0.2)
ΔO_2_Hbi (µmol/l)	3.7 (2.2–6.0)	1.4 (− 0.1 to 3.5)	** < 0.001**	0.70	− 2.2 (− 4.1 to − 0.6)
ΔHHbi (µmol/l)	2.0 (0.3–4.3)	3.8 (1.0 to 5.3)	**0.034**	0.37	1.1 (− 0.6 to 3.2)
ΔcHbi (µmol/l)	6.4 (4.0–9.9)	6.8 (2.9 to 8.8)	0.224	0.21	− 0.4 (− 5.4 to 1.6)
PaO_2_ (mm Hg)	78.0 (73.0–89.5)	90.0 (78.5 to 97.5)	**0.001**	0.57	4.0 (0 to 15.0)
PaCO_2_ (mm Hg)	40.5 (39.0–43.0)	40.5 (38.0 to 43.5)	0.800	0.04	0.5 (− 1.0 to 1.0)
TAPSE (cm)	2.1 (1.9–2.3)	2.0 (1.7 to 2.2)	** < 0.001**	0.68	− 0.2 (–0.3 to 0)
RV diameter (cm)	3.8 (3.6–4.0)	3.9 (3.6 to 4.0)	0.064	0.32	0 (0 to 0.1)

Significant *p* values are given in bold

Data are presented as median and interquartile range. The *p* values were obtained using a paired Wilcoxon signed-rank test, and the effect size was assessed using the rank-biserial correlation (r_rb_) coefficient. For each variable, a difference (Δ) change is provided as the median and interquartile range

*CPP* cerebral perfusion pressure, *ΔcHbi* total hemoglobin, *ΔHHbi* deoxygenated hemoglobin, *ΔO*_*2*_*Hbi*, oxygenated hemoglobin, *ICP* intracranial pressure, *MAP* mean arterial blood pressure, *PaCO*_*2*_ partial pressure of CO_2_, *PaO*_*2*_ partial pressure of O_2_, *PRx* pressure reactivity index, *RM* recruitment maneuvers, *rSO*_*2*_ regional cerebral saturation, *RV* diameter, right ventricle basal diameter, *SpO*_*2*_ systemic oxygen saturation, *TAPSE* tricuspid annulus plane systolic excursion

Changes in cardiopulmonary and echocardiographic variables between T0 and T1 are presented in Table 2. Regarding echocardiographic variables, we observed a reduction in TAPSE (from 2.1 [1.9–2.3] cm to 2.0 [1.7–2.2] cm; *p* < 0.001; Δ = −0.2 cm). Moreover, there was a trend toward RV dilatation after RM (from 3.8 [3.6–4.0] cm to 3.9 [3.6–4.0] cm; *p* = 0.06). Tricuspid regurgitation was observed in only three patients at T0, with an average TRvel of 1.8 cm/s. In these cases, the average TRvel at T1 increased to 2.4 cm/s (statistical analysis not performed as small sample size).

### Correlations between multimodal monitoring parameters as result of RMs

**Fig. 2:**
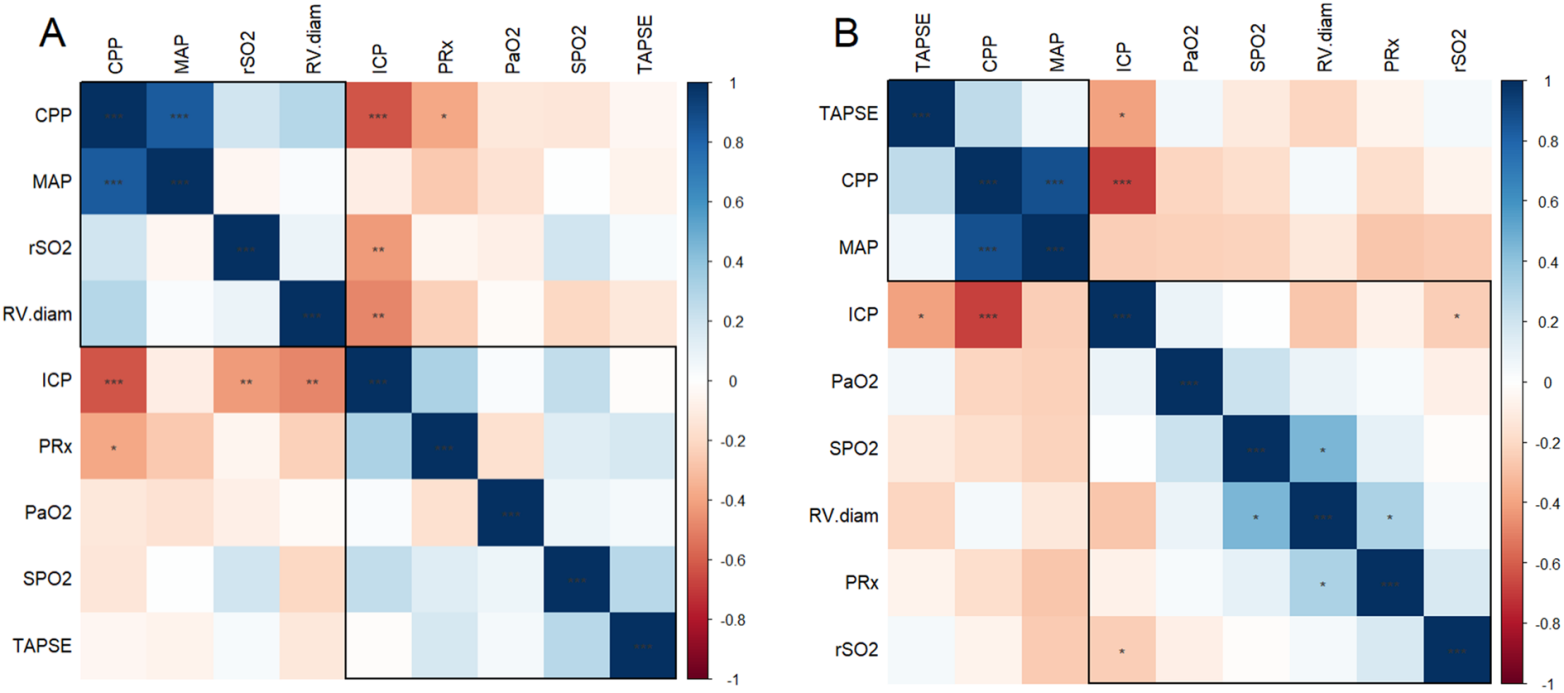
Matrix of Spearman correlation between systemic and neuromonitoring data **A** before (T0) and **B** after (T1) the recruitment maneuvers (RMs). *p* values for Spearman correlation coefficient are marked as follows: ***p < 0.001; **p < 0.01; *p < 0.05. Rectangles around the plot of the correlation matrix are based on the results of hierarchical clustering. *CPP* cerebral perfusion pressure, *ICP* intracranial pressure, *MAP* mean arterial pressure, *PaO*_*2*_ partial pressure of O_2_, *PRx* pressure reactivity index, *rSO*_*2*_ regional cerebral saturation, *RV.diam* right ventricle diameter, *SpO*_*2*_ systemic oxygen saturation, *TAPSE* tricuspid annulus plane systolic excursion

Figure 2 presents the matrix of Spearman correlations in the total group at T0 and T1 ICP exhibited moderate and inverse correlations with both rSO_2_ and RV basal diameter (in both cases,

*r* = −0.48; *p* = 0.005) at T0 (Fig. 2a). CPP also showed a moderate relationship with PRx (*r* = −0.42; *p* = 0.017). Additionally, ICP displayed moderate correlations with rSO_2_ (*r* = −0.39; *p* = 0.039) and TAPSE (*r* = −0.43; *p* = 0.012) (Fig. 2b).

### Subgroup analyses

#### CPP tolerance to RM and changes in monitoring parameters

The CPP tolerance was defined according to values of ΔCPP above (good) or below (poor) the median. At T0, the variables of interest did not differ significantly between poor and good CPP tolerance (Table 3), except for two parameters. The baseline CPP was higher in those with poor tolerance (77.5 [65.9–85.9] mm Hg) as compared with those with good tolerance (65.9 [59.6–73] mm Hg; *p* = 0.040), and the baseline RV diameter was larger in those with poor tolerance (3.9 [3.7–4.0] cm vs. good tolerance 3.6 [3.5–3.9] cm; *p* = 0.034).

**Table 3:** Systemic and neuromonitoring data before and after the recruitment maneuvers (RMs) regarding variation in cerebral perfusion pressure (where ΔCPP was defined as CPP after RM—CPP before RM)

Parameter	Poor CPP tolerance to RMs (*n* = 16)				Good CPP tolerance to RMs (*n* = 16)			
	T0	T1	*p* value	Change	T0	T1	*p* value	Change
ICP (mm Hg)	9.3 (6.1 to 14.7)	18.2 (16.3–21.1)	** < 0.001**	7.8 (3.1 to 11.7)	13.7 (9.1–19.0)	17.4 (12.1–22.8)	0.055	2.8 (0.9 to 5.1)
CPP (mm Hg)	77.5 (65.9 to 85.9)	57.9 (47.5–65.3)	** < 0.001**	− 18.0 (− 20.5 to 14.8)	65.9 (59.6–73)*	65.4 (55.0–73.0)*	0.163	− 3.7 (− 6.1 to 2.6)
MAP (mm Hg)	85.8 (76.9 to 93.6)	77.0 (66.0–80.4)	** < 0.001**	− 9.9 (− 15.6 to − 7.6)	82.5 (75.7–89.7)	83.0 (76.8–88.1)*	0.776	2.0 (− 3.2 to 3.2)
PRx (a.u.)	0.14 (− 0.09 to 0.28)	0.43 (0.41–0.55)	** < 0.001**	0.33 (0.30 to 0.43)	0.30 (0.10–0.37)	0.32 (0.03–0.46)	0.234	0.17 (− 0.30 to 0.36)
SpO_2_ (%)	97.5 (96.5 to 98.0)	98.8 (96.7–99.9)	0.066	1.6 (− 1.0 to 2.0)	97.5 (95.5–98.5)	98.0 (96.5–99.6)	0.388	0.5 (− 1.0 to 2.5)
rSO_2_ (%)	60.9 (55.9 to 64.6)	60.2 (56.1–62.4)	0.163	− 0.3 (− 0.7 to 0.1)	59.0 (52.5–63.4)	58.6 (50.5–63.0)	0.059	–0.8 (–2.0 to 0.3)
PaO_2_ (mm Hg)	78.0 (72.5 to 90.5)	91.5 (82.5–96.0)	**0.012**	7.5 (0.0 to 15.5)	79.0 (75.5–86.0)	87.5 (78.0–97.5)	**0.032**	2.5 (0.0 to 16.0)
PaCO_2_ (mm Hg)	41.0 (39.0 to 44.0)	43.0 (39.0–45.5)	0.331	1.0 (− 1.0 to 1.0)	40.5 (38.5–42.5)	40.0 (36.5–41.5)*	0.552	0.0 (− 2.5 to 1.0)
TAPSE (cm)	2.1 (1.9 to 2.4)	1.9 (1.6–2.3)	**0.002**	− 0.2 (− 0.4 to − 0.1)	2.2 (2.0–2.3)	2.0 (1.9–2.2)	**0.031**	− 0.1 (− 0.3 to 0.0)
RV diameter (cm)	3.9 (3.7 to 4.0)	4.0 (3.8–4.2)	**0.025**	0.1 (0 to 0.2)	3.6 (3.5–3.9)*	3.7 (3.4–3.9)*	0.678	0.0 (0 to 0.1)

Significant *p* values are given in bold

Data are presented as median and interquartile range; analysis was conducted with Wilcoxon signed-rank test for dependent variables

*p* value was obtained using paired Wilcoxon signed-rank test. The comparison of parameters at T0 or at T1 in two subgroups (i.e., T0 poor CPP tolerance vs. T0 good CPP tolerance to RMs) was performed using Mann–Whitney *U*-test. Significant differences were marked as *, which means *p* value < 0.05

*ΔcHbi* total hemoglobin, *ΔHhbi* deoxygenated hemoglobin, *ΔO*_*2*_*Hbi* oxygenated hemoglobin, *ICP* intracranial pressure, *MAP* mean arterial pressure, *PaCO*_*2*_ partial pressure of CO_2_, *PaO*_*2*_ partial pressure of O_2_, *PRx* pressure reactivity index, *RV* diameter, right ventricle diameter, *rSO*_*2*_ regional cerebral saturation, *SpO*_*2*_ systemic oxygen saturation, *TAPSE* tricuspid annulus plane systolic excursion

**Fig. 3:**
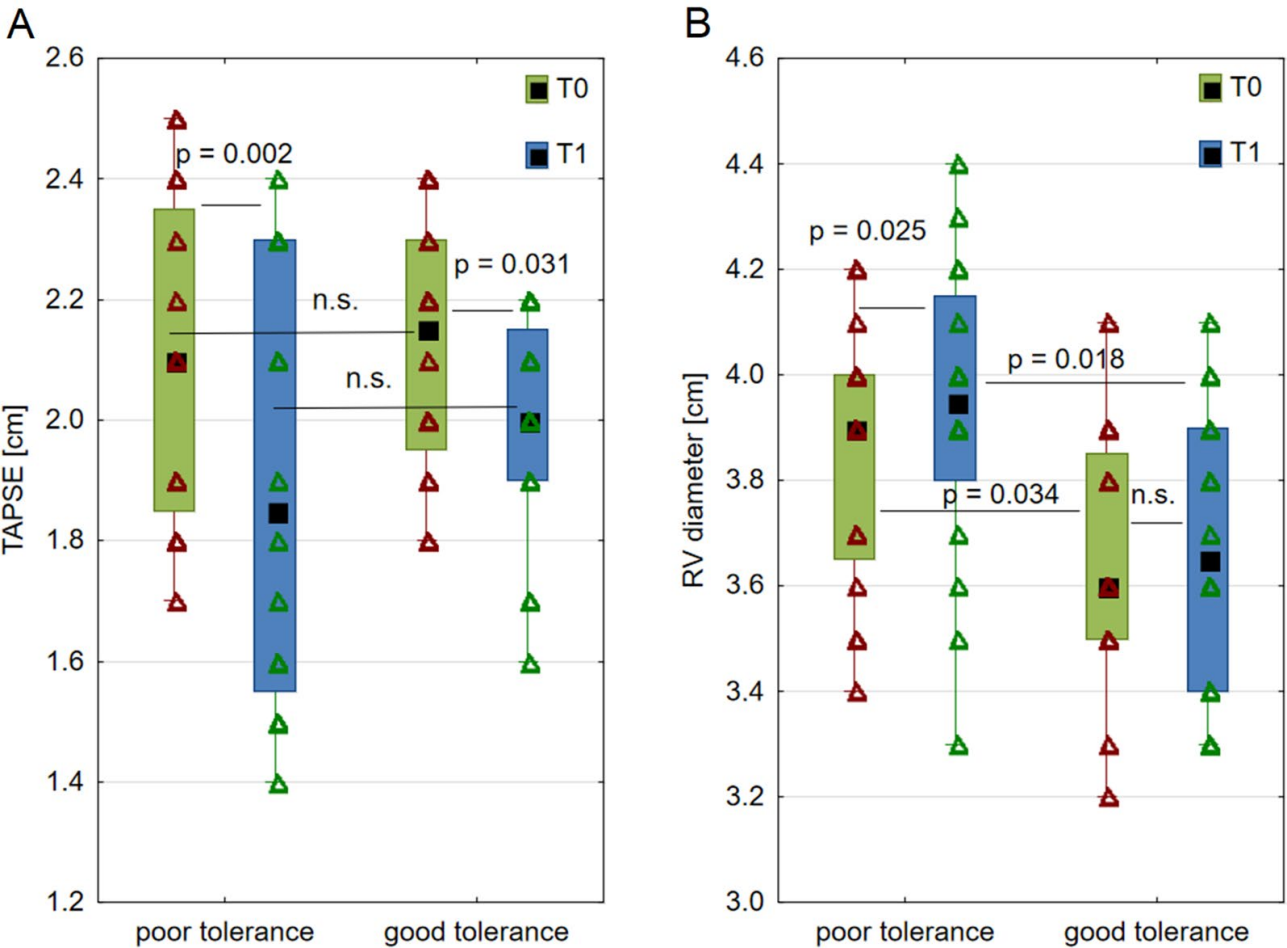
Comparison of **A** tricuspid annulus plane systolic excursion (TAPSE); **B** right ventricle basal diameter (RV diameter); before RMs (T0) and after RMs (T1). Data are presented as median (black squares) and interquartile range; all observations are presented as triangles. *n.s.* not significant, *RM* recruitment maneuver

At T1, the same two variables significantly differed between subgroups, with also significance in difference in MAP and PaCO_2_ (respectively lower and higher in the poor tolerance subgroup). When analyzing differences between T0 and at T1 for each of the two subgroups, we observed several significant changes in the poor tolerance group, whereas only three of these variables (PaO_2_, TAPSE, and ΔO_2_Hbi) significantly changed in the good tolerance group (in all cases in the same direction). Figure 3 shows the changes in TAPSE (Fig. 3a) and RV diameter (Fig. 3b) in both subgroups.

### Correlations according to CPP tolerance to RMs

**Fig. 4:**
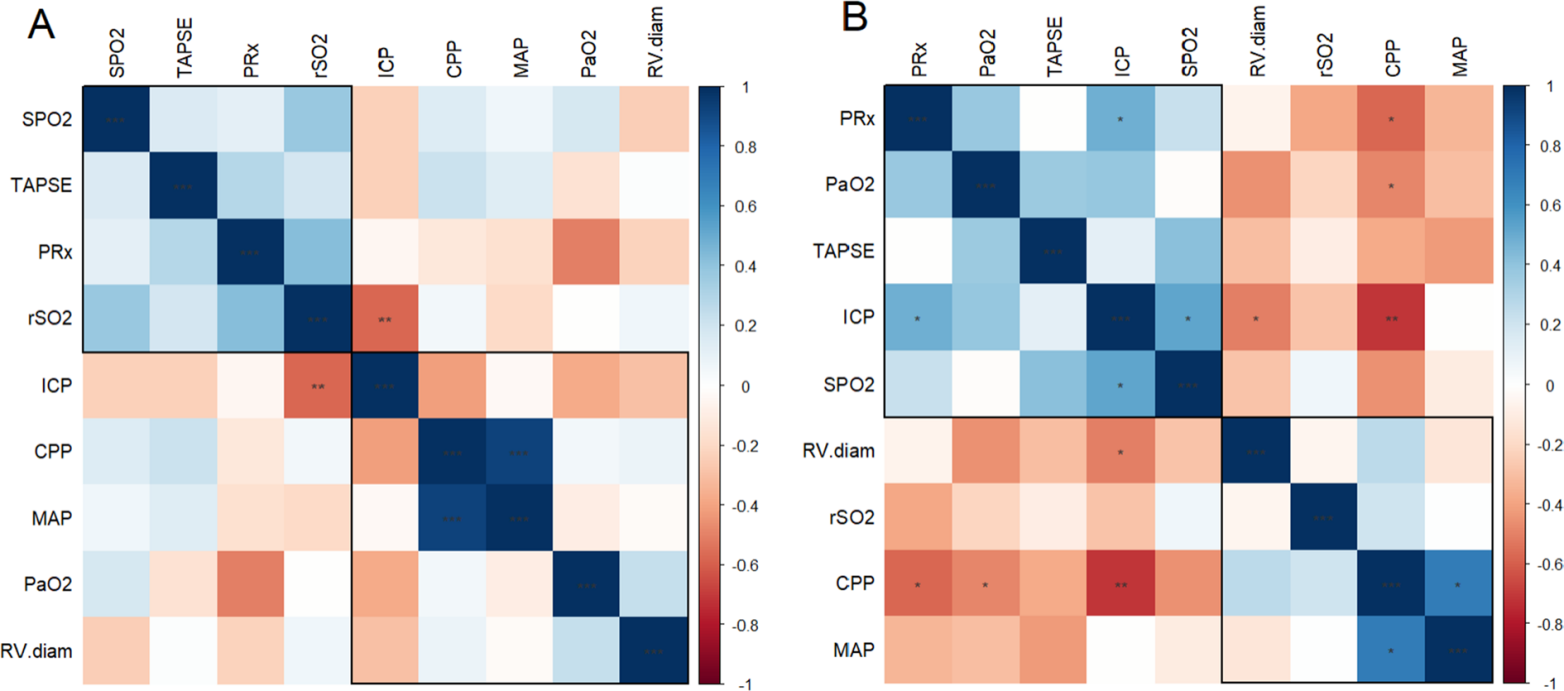
Matrix of Spearman correlation between systemic and neuromonitoring data at baseline (T0) before the recruitment maneuvers (RMs), separating patients with **A** poor CPP tolerance to RMs and **B** good CPP tolerance to RMs. *p* values for Spearman correlation coefficient are marked as: ***p < 0.001; **p < 0.01; *p < 0.05. Rectangles around the plot of the correlation matrix are based on the results of hierarchical clustering. *CPP* cerebral perfusion pressure, *ICP* intracranial pressure, *MAP* mean arterial pressure, *PaO*_*2*_ partial pressure of O_2_, *PRx* pressure reactivity index, *rSO*_*2*_ regional cerebral saturation, *RV.diam* right ventricle diameter, *SpO*_*2*_ systemic oxygen saturation, *TAPSE* tricuspid annulus plane systolic excursion

At T0, the subgroup with poor CPP tolerance to RMs had a significant inverse correlation between ICP and rSO_2_ (*r*_S_ = −0.67, *p* = 0.005), see Fig. 4a. A subgroup with a good CPP tolerance to RMs showed a significant relationship between ICP and systemic oxygen saturation (*r*_S_ = 0.54, *p* = 0.032), and ICP with RV basal diameter (*r*_S_ = −0.58, *p* = 0.019), see Fig. 4b. Moreover, CPP significantly and reciprocal correlated with PRx (*r*_S_ = −0.54, *p* = 0.031), and PaO_2_ (*r*_S_ = −0.53, *p* = 0.035).

### Subgroups analysis according to PRx and ICP after RMs

We conducted further subgroup analyses, providing detailed descriptions of systemic and neuromonitoring data, along with cardiopulmonary and echocardiographic parameters. The initial subgroup analysis involved dividing subgroups based on good and poor PRx tolerance. This classification was defined using ΔPRx values above (indicating poor tolerance) or below (indicating good tolerance) the median ΔPRx value.

Additionally, we conducted two more subgroup analyses, separating groups based on thresholds observed for ICP (22 mm Hg) and PRx (0.3 a.u.) post RMs. All these analyses are presented in the Supplementary material.

## Discussion

In the population of patients with ABI, we observed adverse effects of the RMs on CPP primarily mediated by alterations in its components (ICP and MAP). These effects were clinically significant and remained unaffected by the respiratory impact of RMs, indicated by stable PaCO_2_ values and improved oxygenation. Moreover, we noted a negative influence of RMs on cerebral autoregulation, evidence by an average increase of 0.31 in PRx following RMs. Importantly, the PRx observed post RMs (0.43) exceeded the usual cutoff of 0.30, which typically identifies significant disturbances in cerebral autoregulation.

Notably, rSO_2_ values were marginally decreased (average change − 0.5%) despite higher PaO_2_ values and a trend toward elevated saturation of arterial blood, indicated an increased arterial oxygen content following RMs. Looking at the components of rSO_2_, we observed a significant reduction in its arterial contribution (ΔO_2_Hbi) alongside a simultaneous increase in the venous one (ΔHHbi). Our findings post RMs suggest that cerebral oxygenation was not influenced by the changes in systemic oxygenation (which improved), but mainly by the reduction in CPP and possibly an increase of intrathoracic pressure. Indeed, the decrease in MAP and CPP caused a drop (ΔO_2_Hbi) at cerebral level coupled with an increase in venous oxygen (ΔHHbi) as a possible result of impeded venous return possibly due to congestion from increased intrathoracic pressures. This finding is in line with previous small-scale physiological studies. Mascia et al. [22] studied a cohort of 12 patients with brain injury, in which PEEP was progressively increased. They observed that among patients who positively responded to RMs based on improvements in respiratory mechanics and oxygenation, ICP remained steady. However, in those who did not respond to RMs, ICP significantly increased. The authors also highlighted a substantial correlation between alterations in ICP and pulmonary elastance. On the contrary, according to other authors [23], the primary factor leading to elevated ICP during RMs might be the resultant hypotension induced by the maneuver, emphasizing the necessity to avoid a significant cardiovascular impact during its application. In line with this concept, Nemer et al. [24] suggested that a gradual and gentle application of PEEP during RMs, while ensuring the maintenance of arterial blood pressure and CPP, could facilitate a gradual enhancement of cerebral oxygenation without causing adverse intracranial events.

We hypothesized that the adverse effects on cerebral hemodynamics were potentially attributed to the detrimental impact of RMs on RV function. The physiological increase in intrathoracic pressure induced by RMs may lead to a decline in RV preload and an elevation in PVRs. The preload effect of an RMs might cause venous congestion, leading to jugular vein stasis and subsequently impacting ICP. Additionally, the afterload effects of RMs may further diminish RV stroke volume, consequently reducing left ventricular filling, cardiac output (not measured in our study), and MAP. Moreover, RV dilation itself could hamper left ventricular filling and impair diastole [25, 26]. However, our study is limited by the analysis of only basic echocardiographic variables for the study of the RV.

Furthermore, only in three cases we found a tricuspid regurgitation at baseline, which limited our ability to meaningfully assess the impact of RMs on PVRs. Therefore, we focused on comparing only two basic variables available for assessment: the TAPSE as indicator of RV systolic function and the RV basal diameter representing chamber dilatation. Although we noted a significant difference in TAPSE, it is essential to note that the observed change in RV systolic function may not hold significant clinical meaning. This is because of the small variation and well-known interobserver and intraobserver variability, even among experts [27]. We observed a moderate negative correlation between TAPSE and ICP post RMs, suggesting a potential link between worsened RV longitudinal systolic function and increased ICP. However, in subgroup analyses based on tolerance to RMs concerning CPP and autoregulation, TAPSE decreased significantly in both subgroups. Therefore, TAPSE doesn’t appear to be a useful predictor of RM tolerance. As for the second echocardiographic variable, we noticed a trend toward an increase in RV basal diameter post RM. Interestingly, RV diameter differences at baseline between patients with good or poor CPP tolerance and those showing impaired cerebral autoregulation were evident. Particularly, patients with larger RV diameter at baseline demonstrated poorer tolerance to RMs, suggesting that those with larger RV at baseline may not tolerate RMs well.

Although the observed changes had subtle clinical implications, our findings warrant comprehensive prospective investigations using advanced hemodynamic and echocardiographic analyses to understand the impact of RMs on the RV, especially in patients with borderline or impaired function. Meanwhile, our results provide initial evidence that RMs negatively affect neuromonitoring parameters and MAP independently, indicating a limited major influence on RV function.

Cerebral autoregulatory mechanisms can be disrupted after ABI, thereby impacting blood flow and oxygen delivery. Consequently, cerebral oxygen delivery becomes dependent on CPP [28]. In addition to changes in cerebral autoregulation induced by ABI, RMs can modify arterial partial pressure of oxygen and partial pressure of carbon dioxide in arteries, subsequently influencing the supply of oxygen to nerve cells and impacting ICP. Elevated ICP may lead to inadequate cerebral perfusion, further exacerbating cerebral autoregulatory dysfunction [29].

### Strengths and limitations

Our study has some strengths and several limitations. The main strengths are as follows: the adherence to an established protocol for RMs and the presence of an advanced neurological monitoring beyond the ICP. In addition, this is the first study that describes the interplay between lung, brain, and heart during RM.

However, several limitations should be acknowledged. Firstly, this is a single-center, observational study conducted on patients with ABI over a relatively long enrollment period, encompassing a small number of patients with certain clinical heterogeneity. Despite maintaining an unchanged RM protocol and management of patients with ABI, the extended duration of recruitment might introduce temporal bias. The small sample size in our study potentially introduces biases and restricts the generalizability of our findings. Moreover, considering the heterogeneity between TBI and cerebrovascular diseases (SAH and ICH) within our patient cohort, we acknowledge the potential challenges in combining these subgroups, particularly in analyzing cerebral hemodynamics. Therefore, future larger studies should stratify results according to ABI ethology (TBI, SAH, and ICH). Secondly, a potential selection bias exists as not all patients admitted to our ICU with ABI underwent RMs; only those exhibiting moderate to severe respiratory failure, a worsening trajectory, and showing potential pulmonary benefits from RMs were included. Thirdly, the hemodynamic and echocardiography assessments have been limited to basic variables, and we did not have advanced cardiac output monitoring in place because of the patients’ relatively stable hemodynamics and the low impact of preexisting comorbidities. We also acknowledge that the subgroup analyses according to well-stablished clinical thresholds of ICP (22 mm Hg) and PRx (0.3 a.u.) shown in Supplementary material produced unbalanced subgroups distribution limiting the interpretation of their results. In this context, the definition of poor tolerance was retrospectively determined based on the median delta CPP. Our choice to employ the median as a threshold for poor tolerance was driven by our initial goal of examining the correlation between changes in CPP and the tolerance to the intervention, considering the entire distribution of delta CPP within our study cohort. However, we recognize that this retrospective approach may not offer a clinically standardized assessment of tolerance to interventions, such as RMs.

Finally, cerebral oxygenation data were obtained using NIRS, which, although noninvasive and safe, has significant limitations [30], such as susceptibility to external contamination, unknown sampling area, and mixed blood oxygenated hemoglobin (O_2_Hb) content of unknown proportions. Therefore, caution is advised in interpreting results related to NIRS parameters.

Our results are thought-provoking and should prompt larger and more robust studies. Arguably, the optimal design to comprehend the impact of RMs on cerebral and systemic parameters would involve a cohort study where variables are assessed at baseline and post-RM. It would be advisable to assess the sustainability of the effects induced by RMs by evaluating the measured variables at multiple time points.

Given the potentially negative effects revealed by our study, we advocate for studies employing a more cautious RM approach. Regarding hemodynamic impact, continuous cardiac output monitoring could offer valuable insights into the nuanced changes induced by RMs. Additionally, from an echocardiographic perspective, employing speckle tracking imaging (strain echocardiography) could offer a more detailed depiction of subtle changes.

## Conclusions

In our single-center study, we observed that applying RMs to patients with ABI led to worsened cerebral dynamics, despite demonstrating positive effects on systemic oxygenation. Although the effects of RMs on the RV did not appear to be clinically significant, a larger RV size at baseline might predict poorer CPP tolerance during RMs. Further studies, enrolling a more homogeneous population of patients with neurological injury and evaluating softer approaches to RMs while performing a multimodal neuromonitoring and hemodynamic monitoring approach and exploring association with clinical outcomes are required.

## Supplementary Information

Below is the link to the electronic supplementary material.

Supplementary file1 (DOCX 463 kb)
