# Supplementary material for: The Effect of Recruitment Maneuvers on Cerebrovascular Dynamics and Right Ventricular Function in Patients with Acute Brain Injury: A Single-Center Prospective Study
Source: Neurocrit Care. 2024 Feb 13;41(1):38–48. doi: 10.1007/s12028-024-01939-x (PMC11335957; doi:10.1007/s12028-024-01939-x)
Supplement: Supplementary file 1 — Supplementary file1 (DOCX 463 kb) [file 12028_2024_1939_MOESM1_ESM.docx]

***Subgroups analysis***

***Changes in clinical characteristics regarding PRx after RMs***

At T0, three variables showed significant differences between poor and good PRx tolerance groups (refer to Supplementary Table 1). Specifically, the group with poorer cerebral autoregulation had higher CPP, lower PRx and a larger RV diameter. By T1, the only parameter that remained significantly different between these groups was the RV diameter, once again found to be larger in those with poorer cerebral autoregulation. When analysing differences between T0 and at T1 within each subgroup, several variables had significant changes in both groups: increased ICP and PaO_2_ increased, as well as decreased CPP and TAPSE decreased. However, the subgroup with poorer cerebral autoregulation had a significant increase in PRx and RV diameter, alongside a significant decrease in MAP, changes not observed in the subgroup with better autoregulation. The comparison between subgroups showed that patients with PRx ≥0.3 had significantly lower ICP at T0, along with higher PRx and PaCO_2_ at T1 (refer to Supplementary Table 2). When comparing T0 and T1, only the subgroup with PRx ≥0.3 showed a significant increase in ICP, accompanied by a significant reduction in MAP and CPP, consequently leading to increase in PRx. Moreover, a significant increase in RV diameter and reduction in TAPSE were observed solely in the subgroup with PRx ≥0.3.

***Correlations according to PRx and RMs***

At T0, the subgroup demonstrating poorer cerebral autoregulation (PRx) after RMs exhibited a significant inverse correlation between ICP and two variables: rSO_2_ (r_S_=-0.65, p=0.006) and RV diameter (r_S_=-0.51, p=0.043), as depicted in Supplementary Figure 1A. Conversely, as illustrated in Supplementary Figure 1B, the subgroup with improved cerebral autoregulation after RMs displayed a significant inverse relationship among three pairs of variables [ICP and CPP (r_S_=-0.65, p=0.006), PaO_2_ and RV diameter
(r_S_=-0.55, p=0.029), and TAPSE and MAP (r_S_=-0.51, p=0.042)], as well as a significant positive correlation among four other pairs [MAP and CPP (r_S_=0.57, p=0.022), CPP and rSO_2_ (r_S_=0.51, p=0.043), PRx and SaO_2_ (r_S_=0.62, p=0.010), and TAPSE and PaO_2_ (r_S_=0.59, p=0.016)].

Correlation between systemic and neuromonitoring data at baseline (T0) before RMs, separating patients with PRx≥0.3 and PRx<0.3 is presented in Supplementary Figure 1C-D.

***Changes in clinical characteristics*** ***regarding ICP after RMs***

Patients with ICP ≥22 mmHg had significantly higher ICP and lower CPP at both T0 and T1, along with a smaller RV diameter at T1. A detailed analysis of systemic and neuromonitoring data, cardiopulmonary and echocardiographic parameters, between these two subgroups is presented in Supplementary Table 3 and in Supplementary Figure 2A-B.

**Supplementary Table 1.** Systemic and neuromonitoring data before (T0) and after (T1) the recruitment manoeuvres (RMs) regarding variation in pressure reactivity index (where ΔPRx was defined as PRx after RM – PRx before RM). Data are presented as median and interquartile range; analysis was conducted with Wilcoxon signed rank test for dependent variables.

| Parameter | Poor PRx tolerance to RMs  n = 16 | | |  | Good PRx tolerance to RMs  n =16 | | |  |
| --- | --- | --- | --- | --- | --- | --- | --- | --- |
|  | **T0** | **T1** | **p** | **Δ change** | **T0** | **T1** | **p** | **Δ change** |
| ICP [mmHg] | 10.4  (5.5—13.4) | 16.8  (13.2—18.7) | **0.001** | 4.0  (2.2—9.1) | 15.0  (8.1—19.0) | 19.3  (15.2—22.9) | **0.015** | 5.4  (2.6—6.8) |
| CPP [mmHg] | 78.2  (69.0—85.6) | 64.0  (56,2—71.5) | **0.001** | -16.3  (-19.3— -6.5) | 63.1  (59.0—72.8)* | 57.3  (52.2—68.1) | **0.017** | -5.7  (-14.2— -1.2) |
| MAP [mmHg] | 87.5  (80.2—93.8) | 78.7  (76.0—83.2) | **0.003** | -6.7  (-14.3— -3.3) | 79.5  (74.1—89.7) | 78.5  (68.4—87.0) | 0.111 | -3.1  (-8.0—2.0) |
| PRx [a.u.] | 0.03  (-0.22—0.18) | 0.43  (0.33—0.52) | **<0.001** | 0.39  (0.33—0.44) | 0.33  (0.23—0.42)*** | 0.32  (0.08—0.44) | 0.679 | 0.11  (-0.30—0.20) |
| SpO_2_ [%] | 97.5  (96.5—98.0) | 98.3  (96.5—99.9) | 0.083 | 1.6  (-0.5—2.0) | 97.5  (95.5—99.0) | 98.0  (96.7—100.0) | 0.382 | 0.5  (-1.5—2.5) |
| rSO_2_ [%] | 59.7  (55.8—64.3) | 58.5  (44.8—62.2) | 0.220 | -0.1  (-0.7—0.1) | 61.3  (53.9—63.6) | 59.7  (51.5—62.8) | 0.070 | -0.6  (-2.0—0.3) |
| PaO_2_ [mmHg] | 78.0  (72.5—90.5) | 89.5  (78.5—95.5) | **0.011** | 4.0  (1.5—14.5) | 79.0  (74.0—88.0) | 90.0  (81.0—98.5) | **0.046** | 4.5  (-2.0—20.0) |
| PaCO_2_ [mmHg] | 40.5  (39.0—44.5) | 41.0  (38.0—44.5) | 0.807 | 0.5  (-1.0—1.0) | 40.5  (38.5—42.5) | 40.0  (37.0—43.0) | 0.900 | 0.5  (-1.0—1.0) |
| TAPSE [cm] | 2.2  (1.9—2.4) | 2.0  (1.7—2.3) | **0.004** | -0.2  (-0.3—0.0) | 2.1  (1.9—2.3) | 20  (1.7—2.1) | **0.008** | -0.2  (-0.4— -0.1) |
| RV diameter [cm] | 3.9  (3.7—4.0) | 4.0  (3.9—4.1) | **0.008** | 0.1  (0.0—0.1) | 3.6  (3.5—3.9)* | 3.7  (3.4—3.9)* | 0.767 | 0.0  (-0.1—0.1) |

Abbreviations: ICP, intracranial pressure; MAP, mean arterial pressure; PRx, pressure reactivity index; SaO_2_, systemic oxygen saturation; rSO_2_, regional cerebral saturation; ΔO_2_Hbi, oxygenated haemoglobin; ΔHhbi, deoxygenated haemoglobin; ΔcHbi, total haemoglobin; PaO_2_, partial pressure of O_2_; PaCO_2_, partial pressure of CO_2_; TAPSE, Tricuspid annulus plane systolic excursion; RV diam, right ventricle diameter; p—value was obtained using paired Wilcoxon signed—rank test. The comparison of parameters at T0 or at T1 in two subgroups (i.e. T0 poor PRx tolerance vs T0 good PRx tolerance to RMs) was performed using U Mann-Whitney test. Significant differences were marked as *, which means p-value <0.05

**Supplementary Table 2.** Systemic and neuromonitoring data before (T0) and after (T1) the recruitment manoeuvres (RMs) regarding PRx>0.3 after RMs. Data are presented as median and interquartile range. Analysis was conducted with Wilcoxon signed rank test for dependent variables.

|  | **PRx < 0.3 after RMs**  **(n = 10)** | | | | **PRx ≥0.3 after RMs**  **(n = 22)** | | | |
| --- | --- | --- | --- | --- | --- | --- | --- | --- |
| Parameter | **T0** | **T1** | **p** | **Δ change** | **T0** | **T1** | **p** | **Δ change** |
| ICP [mmHg] | 17.8  (12.9—22.3) | 19.4  (16.7—23.8) | 0.332 | 2.6  (-3.5-6.4) | 10.4  (5.6—14.5)** | 17.4  (12.3—20.2) | **<0.001** | 5.4  (2.9—9.1) |
| CPP [mmHg] | 63.7  (58.9—70.1) | 60.2  (54.2—67.7) | 0.202 | -4.3  (-9.1-2.8) | 72.9  (64.4—85.7) | 62.2  (52.4—71.1) | **<0.001** | -14.4  (-18.5— -6.8) |
| MAP [mmHg] | 84.5  (79.0—90.0) | 79.5  (76.0—88.0) | 0.683 | 0.4  (-8.0-2.0) | 84.5  (74.5—92.2) | 78.5  (69.6—84.0) | **0.002** | -7.6  (-10.5— -3.3) |
| PRx [a.u.] | 0.33  (-0.09—0.43) | 0.08  (-0.0—0.17) | 0.721 | -0.24  (-0.31-0.33) | 0.14  (0.04—0.32) | 0.44  (0.43—0.54)*** | **<0.001** | 0.33  (0.21—0.42) |
| SpO_2_ [%] | 98  (96—99) | 98  (97—100) | 0.612 | 0.5  (0.0—1.0) | 97.0  (95.0—98.0) | 98.3  (96.3—99.8) | 0.082 | 1.3  (-1.0—4.0) |
| rSO_2_ [%] | 58.7  (53.0—64.0) | 57.6  (51.0—62.0) | **0.010** | -1.5  (-3.0— -0.4) | 60.6  (55.6—63.2) | 59.5  (65.0—63.0) | 0.663 | -0.1  (-0.7—0.5) |
| ΔO_2_Hbi [µmol/l] | 4.36  (1.50—6.40) | 0.40  (-0.40—2.30) | **0.017** | -5.11  (-7.0— -2.7) | 3.74  (2.23—5.20) | 2.03  (0.3— 3.70) | 0.485 | -1.9  (-2.8— -0.4) |
| ΔHHbi [µmol/l] | 5.02  (0.64—8.30) | 3.57  (-0.40—6.20) | **0.005** | -2.3  (-3.0—2.9) | 1.82  (0.09—2.58) | 4.0  (1.83—5.05) | **0.008** | 1.4  (0.4—3.3) |
| ΔcHbi [µmol/l] | 9.98  (3.60—15.30) | 6.22  (2.80—9.47) | 0.646 | -7.2  (-9.4— -0.7) | 5.94  (4.40—7.00) | 6.84  (2.93—8.60) | **<0.001** | 0.5  (-1.1—2.2) |
| PaO_2_ [mmHg] | 82.5  (73.0—91.0) | 87.0  (85.0—97.0) | 0.284 | 2.5  (-3.0—10.0) | 78.0  (73.0—89.0) | 91.5  (78.0—98.0) | **0.002** | 5.0  (2.0—16.0) |
| PaCO_2_ [mmHg] | 39.0  (37.0—42.0) | 37.5  (36.0—40.0) | 0.314 | -0.5  (-4.0—1.0) | 41.5  (39.0—44.0) | 42.0  (40.0—44.0)** | 0.276 | 1.0  (-1.0—1.0) |
| TAPSE [cm] | 2.05  (1.90—2.20) | 2.00  (1.90—2.10) | 0.108 | -0.1  (-0.2—0.0) | 2.15  (1.90—2.40) | 2.00  (1.70—2.20) | **<0.001** | -0.2  (-0.3—0.0) |
| RV diam [cm] | 3.55  (3.50—3.90) | 3.60  (3.40—3.90) | 0.989 | 0.0  (0.0-0.1) | 3.85  (3.60—4.00) | 3.90  (3.70—4.10) | **0.033** | 0.05  (0.0-0.1) |

Abbreviations: ICP, intracranial pressure; MAP, mean arterial blood pressure; PRx, pressure reactivity index; SaO_2_, systemic oxygen saturation; rSO_2_, regional cerebral saturation; ΔO_2_Hbi, oxygenated haemoglobin; ΔHhbi, deoxygenated haemoglobin; ΔcHbi, total haemoglobin; PaO_2_, partial pressure of O_2_; PaCO_2_, partial pressure of CO_2_; TAPSE, Tricuspid annulus plane systolic excursion; RV diam, right ventricle diameter; The comparison of parameters at T0 or at T1 in two subgroups (i.e. T0 for PRx <0.3 vs T0 for PRx>0.3) was performed using U Mann-Whitney test. Significant differences were marked as * p-value <0.05; ** p-value <0.01; ***p-value<0.001.

**Supplementary Table 3.** Systemic and neuromonitoring data before and after the recruitment manoeuvres (RMs) test regarding ICP value recorded after RM. Data are presented as median and interquartile range. Analysis was conducted with Wilcoxon signed rank test for dependent variables.

|  | **ICP < 22 mmHg after RMs**  **(n = 23)** | | | | **ICP** ≥**22 mmHg after RMs**  **(n = 9)** | | | |
| --- | --- | --- | --- | --- | --- | --- | --- | --- |
| **Parameter** | **T0** | **T1** | **p-value** | **Δ change** | **T0** | **T1** | **p-value** | **Δ change** |
| ICP [mm Hg] | 9.4  (5.3—12.9) | 16.7  (11.9—18.3) | **0.001** | 3.5  (2.1—9.1) | 17.7  (15.3—19.3)** | 23.4  (22.3—23.9)** | **0.015** | 6.6  (4.5—7.9) |
| CPP [mm Hg] | 76.9  (65.3—85.6) | 65.5  (56.9—71.9) | **0.001** | -13.4  (-18.5—0.7) | 60.5  (58.1—67.7)** | 52.9  (45.4—55.6)** | **0.008** | -9.1  (-14.4— - 4.6) |
| MAP [mm Hg] | 86.5  (79.0—93.0) | 79.3  (76.0—86.0) | **0.006** | -8.0  (-14.4—2.6) | 78.2  (74.5—85.0) | 76.0  (67.4—79.5) | **0.017** | -4.8  (-7.2— -2.0) |
| PRx [a.u.] | 0.13  (-0.07—0.34) | 0.43  (0.23-0.48) | **0.003** | 0.33  (0.19—0.42) | 0.23  (0.23—0.33) | 0.42  (0.23-0.53) | 0.138 | 0.10  (-0.19—0.33) |
| SpO_2_ [%] | 98.0  (95.0—98.5) | 98.0  (96.0—100.0) | 0.217 | 1.0  (-1.0—2.0) | 97.0  (96.0—98.0) | 98.6  (98.0—99.0) | 0.142 | 1.0  (0—2.0) |
| rSO_2_ [%] | 62.0  (57.9—64.0) | 60.0  (56.2—63.0) | 0.121 | -0.1  (-1.0—0.2) | 54.7  (52.0—60.6) | 56.0  (49.9—60.5) | 0.155 | -0.8  (-2.0—0.2) |
| ΔO_2_Hbi [umol/l] | 3.39  (1.84—5.32) | 1.10  (-0.17—2.90) | **<0.001** | -1.7  (-3.6— -0.7) | 5.20  (3.20—6.20) | 2.30  (0.38—7.10) | 0.051 | -2.8  (-6.9— -0.4) |
| ΔHHbi [umol/l] | 1.84  (0.09—4.84) | 4.20  (1.84—5.30) | **0.013** | 1.0  (-0.1—3.3) | 2.30  (1.80—3.42) | 3.50  (-0.20—5.30) | 0.678 | 1.2  (-2.5—1.9) |
| ΔcHbi [umol/l] | 5.68  (3.59—9.80) | 5.30  (2.93—8.90) | 0.377 | -0.4  (-3.2—1.9) | 7.00  (6.60—11.40) | 7.50  (2.80—8.80) | 0.374 | 0.5  (-9.4—1.2) |
| PaO_2_ [mm Hg] | 78.0  (72.0—86.0) | 87.0  (75.0—97.0) | **0.010** | 4.0  (-2.0—16.0) | 87.0  (81.0—90.0) | 94.0  (89.0—98.0) | 0.051 | 4.0  (1..0—10.0) |
| PaCO_2_ [mm Hg] | 42.0  (39.0—44.0) | 42.0  (39.0—44.0) | 0.600 | 1.0  (-1.0—1.0) | 39.0  (38.0—41.0) | 38.0  (36.0—41.0) | 0.709 | 0  (-1.0—1.0) |
| TAPSE [cm] | 2.1  (1.9—2.4) | 2.1  (1.9—2.2) | **0.008** | -0.1  (-0.2—0) | 2.1  (2.0—2.2) | 1.7  (1.6—2.0) | **0.006** | -0.3  (-0.4— -0.2) |
| RV diam [cm] | 3.8  (3.6—3.9) | 3.9  (3.6—4.0) | 0.065 | 0  (0—0.1) | 3.5  (3.5—4.0) | 3.8  (3.4—4.0)* | 0.463 | 0  (0—0.1) |

Abbreviations: ICP, intracranial pressure; MAP, mean arterial blood pressure; PRx, pressure reactivity index; SaO_2_, systemic oxygen saturation; rSO_2_, regional cerebral saturation; ΔO_2_Hbi, oxygenated haemoglobin; ΔHhbi, deoxygenated haemoglobin; ΔcHbi, total haemoglobin; PaO_2_, partial pressure of O_2_; PaCO_2_, partial pressure of CO_2_; TAPSE, Tricuspid annulus plane systolic excursion; RV diam, right ventricle diameter; The comparison of parameters at T0 or at T1 in two subgroups (i.e. T0 for ICP <22 mm hg vs T0 for ICP>22 mm Hg) was performed using U Mann-Whitney test. Significant differences were marked as * p-value <0.05; ** p-value <0.01; ***p-value<0.001.

**
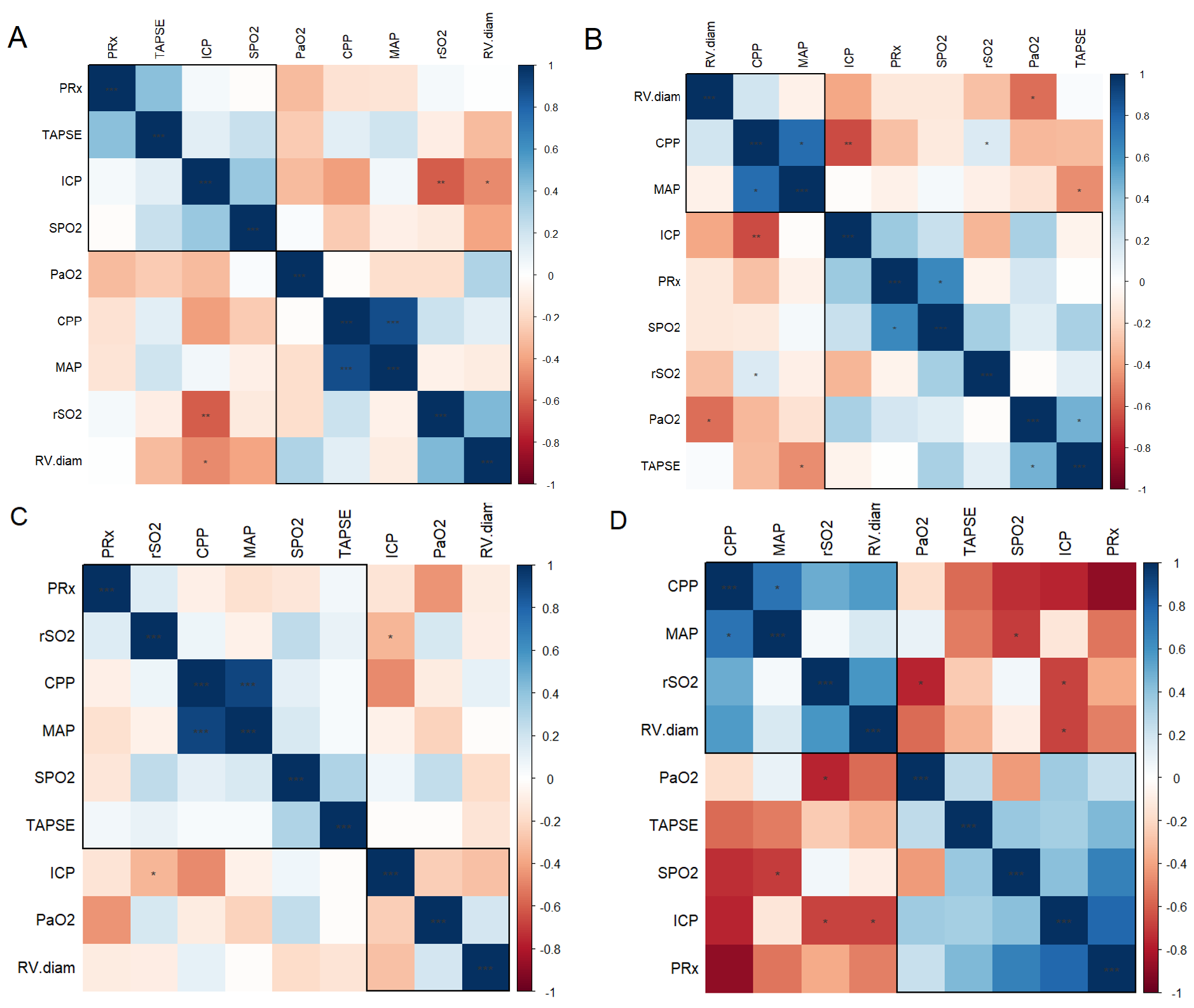
**

**Supplementary Figure 1**. Matrix of Spearman correlation between systemic and neuromonitoring data at baseline (T0) before the recruitment manoeuvres (RMs), separating patients with **A**) poor pressure reactivity index (PRx) tolerance to the RMs and **B**) good PRx tolerance to the RMs; **C**) PRx > 0.3 after RMs and **D**) PRx < 0.3 after RMs; p-values for Spearman correlation coefficient are marked as: *** p < 0.001; ** p < 0.01; * p < 0.05. Rectangles around the plot of the correlation matrix are based on the results of hierarchical clustering. Abbreviations: ICP, intracranial pressure; MAP, mean arterial pressure; CPP, cerebral perfusion pressure; PRx, pressure reactivity index; SpO_2_, systemic oxygen saturation; rSO_2_, regional cerebral saturation; PaO_2_, partial pressure of O_2_; TAPSE, Tricuspid annulus plane systolic excursion; RV.diam, right ventricle diameter.

**
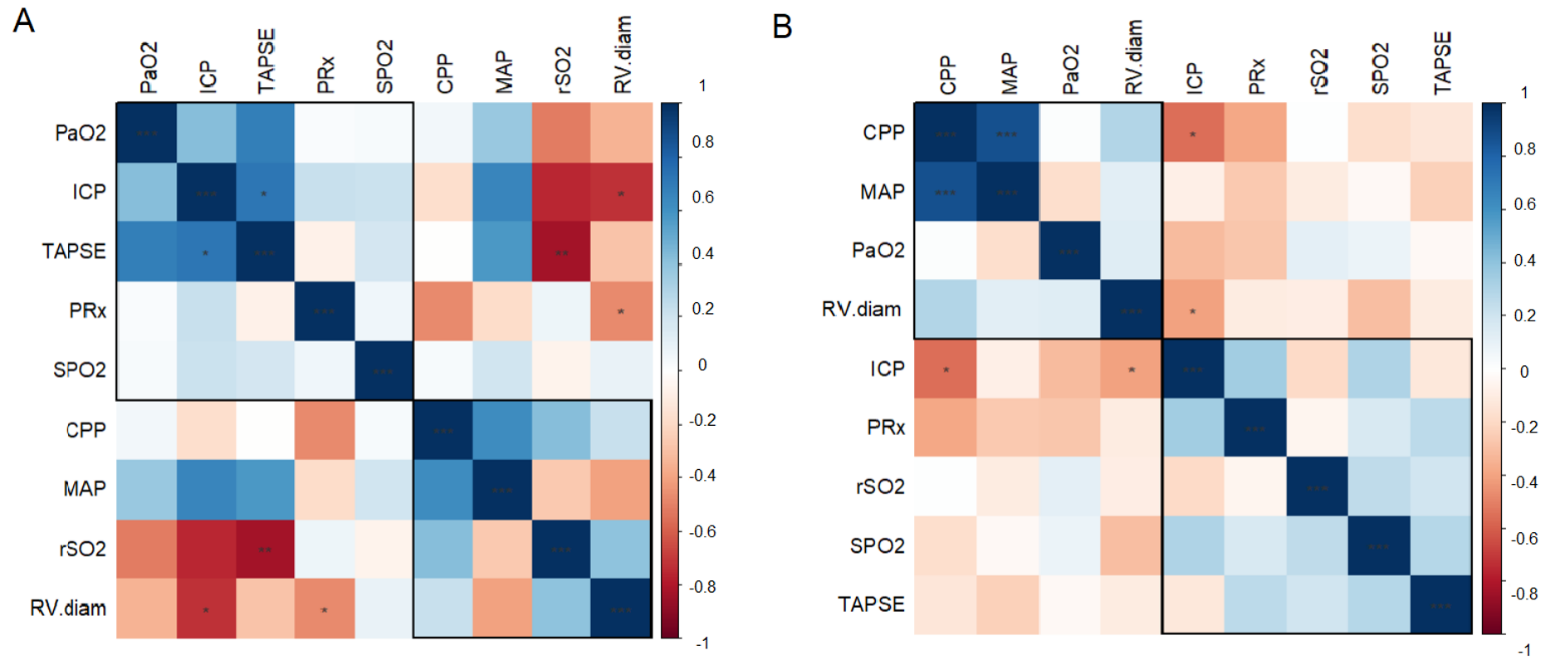
**

**Supplementary Figure 2.** Matrix of Spearman correlation between systemic and neuromonitoring data at baseline (T0) before the recruitment manoeuvres (RMs) in patients with (**A**) intracranial pressure (ICP)> 22 mm Hg after RMs and (**B**) ICP < 22 mm Hg after RMs; p-values for Spearman correlation coefficient are marked as: *** p < 0.001; ** p < 0.01; * p < 0.05. Rectangles around the plot of the correlation matrix are based on the results of hierarchical clustering. Abbreviations: ICP, intracranial pressure; MAP, mean arterial pressure; CPP, cerebral perfusion pressure; PRx, pressure reactivity index; SpO_2_, systemic oxygen saturation; rSO_2_, regional cerebral saturation; PaO_2_, partial pressure of O_2_; TAPSE, Tricuspid annulus plane systolic excursion; RV.diam, right ventricle diameter.
